# Improvements and Disparities in Types of Foods and Milk Beverages Offered in Elementary School Lunches, 2006–2007 to 2013–2014

**DOI:** 10.5888/pcd13.150395

**Published:** 2016-03-17

**Authors:** Lindsey Turner, Punam Ohri-Vachaspati, Lisa Powell, Frank J. Chaloupka

**Affiliations:** Author Affiliations: Punam Ohri-Vachaspati, School of Nutrition and Health Promotion, Arizona State University, Phoenix, Arizona; Lisa Powell, Frank J. Chaloupka, Institute for Health Research and Policy, University of Illinois at Chicago, Chicago, Illinois.

## Abstract

**Introduction:**

Children consume much of their daily energy intake at school. School district policies, state laws, and national policies, such as revisions to the US Department of Agriculture’s school meals standards, may affect the types of foods and beverages offered in school lunches over time.

**Methods:**

This study evaluated changes and disparities in school lunch characteristics from 2006–2007 to 2013–2014. Data were obtained from annual cross-sectional surveys at 4,630 public elementary schools participating in the National School Lunch Program. Multivariate logistic regressions were conducted to examine lunch characteristics.

**Results:**

The percentage of schools regularly offering healthful items such as vegetables (other than potatoes), fresh fruit, salad bars, whole grains, and more healthful pizzas increased significantly from 2006–2007 to 2013–2014, and the percentage of schools offering less healthful items such as fried potatoes, regular pizza, and high-fat milks decreased significantly. Nevertheless, disparities were evident in 2013–2014. Schools in the West were significantly more likely to offer salad bars than were schools in the Northeast, Midwest, or South (adjusted prevalence: West, 66.3%; Northeast, 22.3%; Midwest, 20.8%; South, 18.3%). Majority-black or majority-Latino schools were significantly less likely to offer fresh fruit than were predominantly white schools (adjusted prevalence: majority black, 61.3%; majority Latino, 73.0%; predominantly white, 87.8%). Schools with low socioeconomic status were significantly less likely to offer salads regularly than were schools with middle or high socioeconomic status (adjusted prevalence: low, 38.5%; middle, 47.4%; high, 59.3%).

**Conclusion:**

Much progress has been made in improving the quality of school lunches in US public elementary schools, but additional opportunities for improvement remain.

## Introduction

Many US children consume diets that exceed recommended amounts of sugar and fat (1,2) and are inadequate in fruits, vegetables, and whole grains (3,4). Major caloric contributors to children’s diets include sugar-sweetened beverages (SSBs), desserts, pizza, and whole-fat milks (5,6). Between 1994 and 2010, the percentage of children’s energy intake from sugar and fat obtained at school improved, but flavored milks, fried potatoes, and pizza consumed at schools still significantly contributed to empty calories in 2009–10 (1).

School meal programs play a crucial role in shaping children’s diets (7,8); one-third to one-half of children’s daily energy intake is from foods and beverages consumed during the school day (8). Switching from SSBs and flavored milks to unflavored low-fat milks at school and home would save elementary-aged children an estimated average of 173 calories per day (9). Data gathered in 2004–2005 showed that most schoolchildren, particularly those who participated in school meals programs, consumed too much saturated fat and sodium (10).

Most US public schools participate in the National School Lunch Program (NSLP) (11), which provides meals to approximately 31 million students annually (11), and therefore has enormous reach and impact on the healthfulness of options available to students during the school day. Although meal standards are established by the US Department of Agriculture (USDA), each local school food authority decides what to serve. As directed by the Healthy, Hunger-Free Kids Act of 2010, the USDA released new standards to be implemented by the start of 2012–2013 (12). Key changes were the requirement that both a fruit *and* a vegetable be offered each day and that a variety of types of vegetables be offered within each week, including groups such as dark green and red or orange. Milk was limited to nonfat or low-fat (1%) milk, and sweetened flavored milk is now allowed only if nonfat. Half of grains offered were to be whole-grain–rich by 2012–2013, increasing to all whole-grain–rich by the 2014–2015 school year, although this deadline was delayed until 2015–2016 (13). The provisions on fat content may have affected fried potatoes and pizza, and some schools reformulated pizzas to be more healthful by using lower-fat cheese than previously used, vegetables instead of meat toppings, and whole-grain crusts. Recent reports show substantial improvements in school lunches in elementary and secondary schools (14,15); one study documented reductions in socioeconomic disparities in lunch characteristics among secondary schools, with more rapid improvements at smaller schools, and significant improvements at schools serving a high proportion of nonwhite students (15). Nevertheless, demographic and geographic disparities have not yet been examined thoroughly at elementary schools. Given research showing more healthful food environments at schools in the Pacific census division (California, Oregon, and Washington) during 2009–2010 (16) and the relatively early engagement of western states such as California in farm-to-school programming (17), which has been associated with a higher prevalence of salad bars in schools (18), we examined potential regional, socioeconomic, and demographic disparities.

This study examined changes in lunches at nationally representative samples of US public elementary schools from 2006–2007 to 2013–2014. Of particular interest were changes over time; disparities by region, locale, and student demographics; and the extent to which disparities persisted in 2013–2014. Such data can indicate which schools may benefit from additional targeted assistance.

## Methods

Data were gathered as part of the Bridging the Gap research program, a large multiyear project that examined school wellness–related policies and practices. This study used data from the Food and Fitness elementary school survey, which was conducted annually from 2006–2007 to 2013–2014 in nationally representative samples of schools. An overview is provided below; details are available elsewhere (19). The study was approved by the institutional review board at the University of Illinois at Chicago, with a waiver of written documentation of consent; consent was implied by mailing back the survey.

### Sampling and weighting

Annual samples were based on sampling frames developed from the public-use Common Core of Data from the National Center for Education Statistics. All public elementary schools in the contiguous United States and the District of Columbia that had a 3rd grade class with at least 20 students were eligible for sampling. Annual survey response rates using standard calculations (20) were 54.6%, 70.6%, 61.8%, 64.5%, 57.4%, 53.3%, 59.3%, and 61.2%, respectively, across the 8 school years. Weights were developed that allowed for inference to US elementary schools; calibration adjusted for potential nonresponse bias.

Surveys were mailed to schools annually in January; a $100 incentive was offered for completion. Follow-up by mail, email, and telephone occurred until July of each year. Instructions requested that the principal complete items on school-wide practices and policies and that a food service professional (FSP), if the school had one, complete the food and beverage items. At most schools, the entire survey was completed by principals; FSPs completed surveys at 30% of schools. Analyses controlled for whether a FSP was involved or not.

### Measures

The survey was developed by researchers in 2006–2007 to be consistent with items already used by the Bridging the Gap middle and high school survey project. In addition, a research review was used in survey development; surveys were tested with experts to confirm face validity.

**Lunch characteristics.** Respondents were asked to indicate how often each of a list of foods and milk beverages was available to elementary students in “the school lunch meal (not à la carte),” with response options of 1 (never), 2 (some days), or 3 (most or every day). These items included vegetables (excluding potatoes), fresh fruit, other fruit (eg, dried or canned fruit), salad bar, premade main-course salads (such as chef’s salad), whole grains (such as wheat bread or brown rice), and fried potatoes (including reheated french fries or tater tots). Starting in 2010–2011, respondents were asked to indicate whether the school offered “regular” pizza or “healthier” pizza (eg, whole-wheat crust, low-fat cheese, or low-fat toppings). Healthful items were combined as binary variables to compare “most or every day” versus “some days or never,” whereas less healthful options (fried potatoes and regular pizza) were combined to compare responses of “some days, most days, or every day” versus “never.” Because some schools had difficulty implementing the whole-grain provisions, a second approach was also used for whole grains; this approach compared “most or every day, or some days” with “never.” Analyses showed that some schools offered salad bars, whereas others offered premade salads, so a new item was calculated to indicate regular availability of any salad (either type). A series of items asking about milk beverages at various levels of fat content and flavoring were combined to indicate whether any high-fat milks (ie, 2% or whole-fat, unflavored or flavored) were ever offered. Although the new regulations do not allow 1% milk if flavored (only nonfat flavored milk is allowed), information on milk items was not detailed enough in the early years of the project to make this distinction. Thus, the current analyses considered only 2% and whole milk (not 1% flavored milk) as high-fat milk.

**Contextual factors.** School-level demographic data were obtained from public-use files from the National Center for Education Statistics for use as covariates in regression analyses. Demographic variables were the following: region (South, West, Midwest, or Northeast); locale (urban, suburban, town, or rural); school size (small, ≤450 students; medium, 451–621 students; or large, ≥622 students); percentage of students eligible for free or reduced-price lunch (FRPL) as an inverse proxy for socioeconomic status (SES) (low, ≤33% eligible; middle, >33% but ≤66% eligible; high, >66% eligible), and racial/ethnic composition (predominantly [≥66%] white non-Latino, majority [≥50%] black non-Latino, majority Latino, and “other”). The “other” category included schools with diverse populations and no majority group and schools with majority Asian or majority American Indian students.

### Data analysis

The initial data set were from 5,068 schools. Because this study focused on implementation of USDA standards, only schools participating in the NSLP (93.6%) were included. Of the 4,745 schools participating in the NSLP, 115 did not provide data on lunches and were excluded, leaving data from 4,630 schools for analysis. The data were treated as repeated (stacked) cross-sections; analyses were conducted in Stata/SE version 12.0 (StataCorp LP) and accounted for sampling stratum and for the clustering of schools within districts. Data were weighted to provide inference to US public elementary schools.

Analyses (conducted in a multivariate logistic regression framework, controlling for year, contextual covariates, and whether an FSP participated) examined disparities and changes over time for each of the 12 outcomes. First, a series of multivariate logistic regressions examined changes over time. Adjusted prevalences (the percentage of schools where each item was available), controlling for covariates, were calculated. Next, a series of demographic-by-time interactions was tested, using a linear trend for time multiplied separately by each demographic variable. Finally, disparities during the 8 years (ie, main effects for contextual covariates) and in the 2013–2014 school year only were examined.

## Results

The sample included a diverse cross-section of schools (Table 1); demographics did not differ by year, except for FRPL eligibility: the percentage of schools at which more than two-thirds of students were eligible for FRPL increased from 28.9% in 2006–2007 to 37.7% in 2013–2014 (consistent with national increases [21]).

**Table 1:** Demographic Characteristics of 4,630 US Public Elementary Schools That Provided Data on School Lunch Practices, School Years 2006–2007 to 2013–2014^a^

Characteristic	Percentage^b^
**Region^c^**	
South	35.7
West	23.3
Midwest	25.0
Northeast	16.0
**Locale**	
Urban	32.6
Suburban	28.8
Town	11.5
Rural	27.1
**School size**	
Small (≤450 students)	47.8
Medium (451–621 students)	30.6
Large (≥622 students)	21.6
**School socioeconomic status^d^**	
High (≤33% eligible)	26.5
Middle (>33% to ≤66% eligible)	38.3
Low (>66% eligible)	35.2
**Student race/ethnicity**	
Predominantly (≥66%) white non-Latino	45.2
Majority (≥50%) black non-Latino	10.6
Majority (≥50%) Latino	17.9
Other^e^	26.3

a We found no significant differences in school demographic characteristics between 2006–2007 and 2013–2014, except percentage of students eligible for free or reduced-priced meals, which increased.

b Percentages are weighted to the school level.

c The 48 noncontiguous states and the District of Columbia were grouped into the following census regions: South (Alabama, Arkansas, Delaware, District of Columbia, Florida, Georgia, Kentucky, Louisiana, Maryland, Mississippi, North Carolina, Oklahoma, South Carolina, Tennessee, Texas, Virginia, West Virginia); West (Arizona, California, Colorado, Idaho, Montana, Nevada, New Mexico, Oregon, Utah, Washington, Wyoming); Midwest (Illinois, Indiana, Iowa, Kansas, Michigan, Minnesota, Missouri, Nebraska, North Dakota, Ohio, South Dakota, Wisconsin); Northeast (Connecticut, Maine, Massachusetts, New Hampshire, New Jersey, New York, Pennsylvania, Rhode Island, Vermont).

d Percentage of students eligible for free or reduced-price lunch was used as an inverse proxy for socioeconomic status.

e “Other” category comprises schools with diverse populations and no majority group and schools with majority Asian or American Indian students.

The availability of vegetables increased significantly from 2008–2009 to 2013–2014 (Table 2); we found no time-by-demographic interactions or disparities in availability.

**Table 2:** Adjusted Percentages^a^ of Public Elementary Schools Offering Selected Food and Beverage Items in School Lunches, by School Year

Item	2006–2007	2007–2008	2008–2009	2009–2010	2010–2011	2011–2012	2012–2013	2013–2014	P Value for Linear Trend
**More healthful items^b^**									
Vegetables	—	—	75.0	78.3	81.3	83.9	85.3	85.5	<.001
Fresh fruit	60.9	61.1	61.4	66.5	68.5	75.1	76.8	81.6	<.001
Other fruit	40.6	45.2	40.2	48.2	44.4	42.6	44.3	50.9	.03
Salad bar	16.4	20.9	21.7	20.6	20.3	29.8	28.7	31.5	<.001
Premade salad	24.7	24.3	21.7	22.7	26.7	30.0	26.0	24.6	.28
Any salad (salad bar or premade)	36.1	38.2	38.2	37.3	40.0	49.8	47.1	47.1	<.001
Whole grains	14.6	21.2	20.8	22.9	41.5	41.4	49.6	48.6	<.001
Ever offers whole grains^c^	76.6	83.2	83.0	88.8	90.3	95.8	97.5	97.2	<.001
Ever offers more healthful pizza^c^^, ^ d	—	—	—	—	64.1	72.8	84.8	88.1	<.001
**Less healthful items^c^**									
Regular pizza	—	—	—	—	70.4	63.1	47.1	34.7	<.001
Fried potatoes	—	—	73.5	72.8	70.5	64.0	60.0	53.1	<.001
High-fat (2% or whole fat) milk	78.3	79.0	70.5	64.7	56.7	44.2	40.4	29.0	<.001

Abbreviation: —, items were not included in survey in given year.

a Adjusted for covariates, including region, locale, school size, racial/ethnic composition of students, school socioeconomic status (percentage of students eligible for free or reduced-priced meals), and whether a food service professional (rather than, for example, the school principal) answered the questions on food and beverage items available.

b Available on most days or every day, unless otherwise noted.

c Available on some days, most days, or every day.

d More healthful pizza was defined as pizza that had, for example, whole-wheat crust or lower-fat cheese or toppings than regular pizza.

The availability of fresh fruit increased significantly from 60.9% in 2006–2007 to 81.6% in 2013–2014, with no time-by-demographic interactions. We found several disparities in fresh fruit availability, which remained significant in 2013–2014. In 2013–2014, predominantly white schools were significantly more likely to offer fresh fruit (adjusted prevalence, 87.8%; 95% confidence interval [CI], 83.5%–92.1%) than were majority-black schools (61.3%; 95% CI, 44.9%–77.8%; *P* = .002) or majority-Latino schools (73.0%; 95% CI, 60.9%–85.1%; *P* = .03) but not more likely than schools with “other” composition (82.7%; 95% CI, 76.3%–89.1%). Multivariate models indicated that schools in urban locales were significantly more likely to offer fresh fruit in 2013–2014 (89.0%; 95% CI, 83.9%–94.0%) than were schools in towns (74.1%; 95% CI, 62.5%–85.8%; *P* = .01) or rural locales (73.5%; 95% CI, 63.9%–83.1%; *P* = .01) but not suburban locales (82.9%; 95% CI, 77.1%–88.6%).

**Figure 1:**
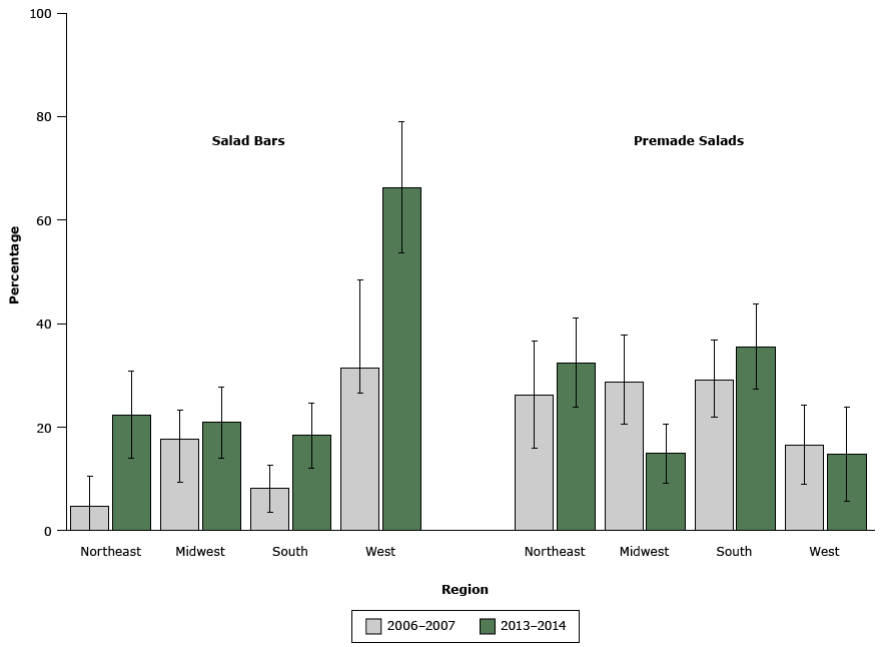
The percentage of elementary schools in the United States offering salad bars or premade salads at lunch, by region, 2006–2007 (n = 524) and 2013–2014 (n = 596). Error bars indicate 95% confidence intervals. **Table:** 

The availability of salad bars increased significantly from 16.4% in 2006–2007 to 31.5% in 2013–2014. Although the time-by-region interaction was not significant, we found a regional disparity, which remained in 2013–2014 (Figure 1). In 2013–2014 the availability of salad bars was significantly higher in the West than in all other regions (*P* <.001 for all comparisons).

Type of Salad, by Region	2006–2007, % (95% Confidence Interval)	2013–2014, % (95% Confidence Interval)
Salad bars		
Northeast	4.6 (0.0–10.3)	22.3 (13.9–30.7)
Midwest	17.6 (9.3–23.2)	20.8 (13.9–27.6)
South	8.1 (3.5–12.5)	18.3 (11.9–24.6)
West	31.3 (26.4–48.4)	66.3 (53.6–79.0)
Premade salads		
Northeast	26.2 (15.9–36.6)	32.4 (23.7–41.0)
Midwest	28.6 (20.4–37.7)	14.8 (9.1–20.5)
South	29.1 (21.8–36.7)	35.4 (27.2–43.7)
West	16.4 (8.8–24.2)	14.7 (5.5–23.8)

The availability of premade salads did not change from 2006–2007 (24.7%) to 2013–2014 (24.6%), but the time-by-region interaction was significant (*P* = .02); availability decreased in the Midwest, increased in the Northeast and South, and did not change in the West (Figure 1). 

We found a significant disparity in the availability of any salad (salad bar or premade) among schools by SES status. Across all years, low-SES schools had less availability of any salad than did middle-SES or high-SES schools (*P* <.001 for both comparisons), and in 2013–14, availability of any salad was significantly lower in low-SES schools (38.5%; 95% CI, 29.8%–47.1%, *P* = .005) than in high-SES schools (59.3%, 95% CI, 49.5%–69.1%) (*P* = .005) and lower (but not significantly) than in middle-SES schools (47.4%; 95% CI 39.7%–55.0%, *P* = .053).

The availability of whole grains increased significantly from 14.6% in 2006–2007 to 48.6% in 2013–2014; no time-by-demographic interactions were found. In 2013–2014, no demographic disparities were found.

The prevalence of schools ever offering more healthful pizzas increased from 64.1% in 2011–2012 to 88.1% in 2013–2014, while the availability of regular pizza decreased. No time-by-demographic interactions or disparities were found in 2013–2014.

The percentage of schools ever offering fried potatoes decreased significantly from 73.5% in 2008–2009 to 53.1% in 2013–2014; no time-by-demographic interactions or disparities were found in 2013–2014.

**Figure 2:**
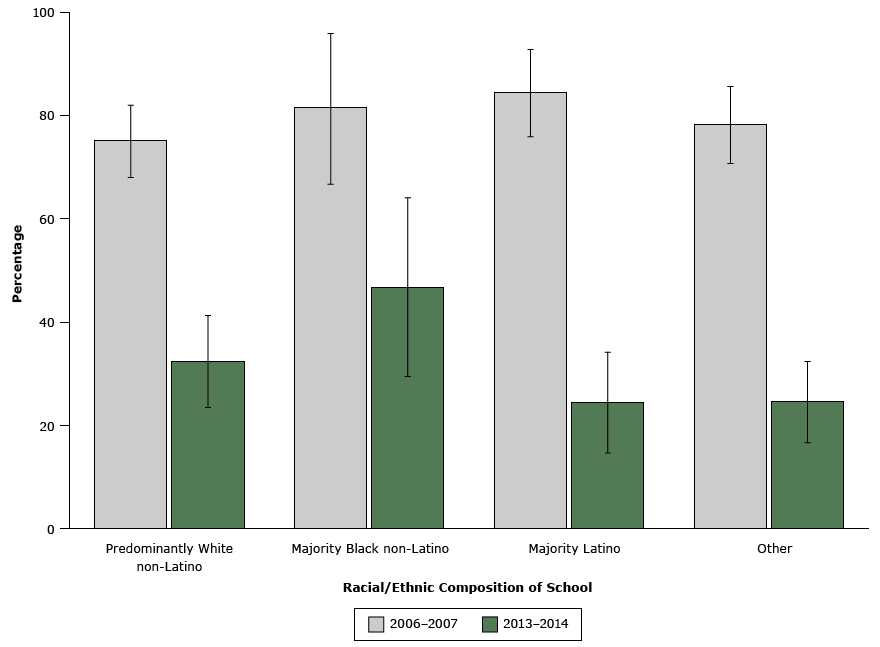
The percentage of elementary schools in the United States offering high-fat milks (2% or whole milk) at lunch, by student race/ethnicity, 2006–2007 (n= 524 schools) and 2013–2014 (n = 596 schools). Predominantly white non-Latino was defined as ≥66% white non-Latino; majority black non-Latino, ≥50% black non-Latino; majority Latino, ≥50% Latino; and “other.” Error bars indicate 95% confidence intervals. **Table:** 

The availability of high-fat milks decreased, and we found a significant interaction between time and student race/ethnicity (*P* = .04). Availability decreased significantly among all racial/ethnic groups, but the decrease was slower at majority-black schools (Figure 2). Although the prevalence of high-fat milks was higher at majority-black schools in 2013–2014 than at predominantly white, majority Latino, and “other” schools, the difference was not significant.

Racial/ethnic composition of school	2006-2007, % (95% Confidence Interval)	2013-2014, % (95% Confidence Interval)
Predominantly white non-Latino	75.1 (68.0–82.0)	32.4 (23.5–41.3)
Majority black non-Latino	81.6 (66.7–95.9)	46.8 (29.5–64.1)
Majority Latino	84.5 (75.9–92.8)	24.5 (14.7–34.2)
Other	78.3 (70.7–85.6)	24.6 (16.7–32.4)

## Discussion

Using data gathered during 8 consecutive school years — during a time of policy action at many levels and nationwide revisions to NSLP standards — our study examined changes in the types of foods and milk beverages offered in elementary school lunches. Overall, the changes were positive: the availability of vegetables, fresh fruit, salad bars, whole grains, and more healthful pizzas increased, while the availability of high-fat milks, fried potatoes, and regular pizza decreased.

A key finding was the increase in the overall availability of salad bars, but we found important regional differences. In the Midwest, the proportion of schools offering premade salads decreased, whereas the proportion offering salad bars slightly increased. Schools in the South were more likely than other regions to offer premade salads and were less likely to offer salad bars. One interpretation of this pattern is that schools in the South may be turning to premade salads as a strategy to increase student access to fresh fruits and vegetables. Although no evidence suggests that premade salads are nutritionally inferior to salad bars, some evidence suggests that salad bars can increase students’ fruit and vegetable consumption at lunch (22,23). Research is needed on the nutritional composition of salad bars and the contribution of salad toppings and dressings to fat and sodium consumption. Although the cost of new equipment for salad bars can be a barrier to implementation, financial support is available through programs such as Let’s Move Salad Bars to School, which provides financial resources and technical support to implement salad bars, especially in low-SES schools (24). Low-SES schools had less availability of any salad than did middle-SES or high-SES schools across all survey years; in 2013–2014, low-SES schools had the least availability of any salads, showing that continued implementation support and resources are needed.

The prevalence of obesity among children could be decreased by switching from SSBs and flavored milks to low-fat milk with meals (9). Our findings on racial/ethnic disparities in milk offerings are consistent with dietary intake data showing that black children are more likely than white children to consume high-fat milk at school (25). The smaller decreases in the prevalence of high-fat milk in majority-black and majority-Latino schools compared with predominantly white schools could reflect the perceptions among food service personnel of a reluctance among black and Latino students to consume low-fat milk. Data from 17 public school districts, however, showed that although NSLP participation rates dropped when low-calorie flavored milk was first substituted for regular-fat flavored milks, participation rates recovered (26).

Although children cannot make healthful choices if none are available, simply offering more healthful items may be insufficient to change behavior. In addition to changes in offerings, promotion and marketing interventions (eg, taste tests, social marketing) may be needed. Such interventions increased the consumption of whole grains (27,28) and fruit and vegetables (22) among students.

The whole-grains provision of the new NSLP standards has been challenging for school meal programs, and the deadline for meeting the provision was extended (13) to allow school food service programs and manufacturers time to identify suitable grain products. In 2013–2014, nearly all schools (97.2%) offered whole grains on some days, most days, or every day of the week, whereas only half (48.6%) offered whole grains on most days or every day. The wording of our survey items did not allow for detailed evaluation of the types of whole-grain–rich items offered, and some items may not have been considered by survey respondents as whole grain, which is defined by USDA as 100% whole grain or at least 50% whole grain with the remaining grains being enriched (29). Menu analysis and site audits are preferable to surveys for such detailed analyses.

We cannot make clear assertions on the causes of improvements: even before the new standards, many districts and states had improved school meals. The prevalence of fresh fruit and salad bars increased between 2010–2011 and 2011–2012, possibly in anticipation of the new standards. Many districts developed local wellness policies in 2006–2007, and although many wellness policies started out weak and fragmented, the comprehensiveness and strength of meals provisions have since increased (30). Although the USDA meal regulations were not announced until 2012, they were based largely on recommendations from the Institute of Medicine (7) in 2009. School food authorities may have started to implement these changes prior to the revised USDA standards. Our data suggest that improvements in lunches were under way before the deadline for nationwide implementation, illustrating the importance of policy actions at multiple levels for promoting school-level change.

Our study has several limitations. Survey data are subject to social desirability bias or lack of knowledge among respondents. Although analytic weights were adjusted for potential nonresponse bias, other factors may have systematically biased which schools responded. If such biases were related to school practices — for example, if schools with better practices were more likely to respond — then our estimates would be biased. Although we do not have any evidence of response bias related to meal quality, we cannot rule out the possibility that schools with more nutritious meals were more likely to respond. Although we requested that food service personnel complete the lunch items, many respondents were administrators, who may have been less familiar with lunches than food service staff would have been. It is our experience that many elementary school principals spend enough time in the cafeteria to be able to accurately provide general information about the presence of salad bars or the types of foods and beverages available to students at lunch; however, some questions — such as those on whole-grain–rich foods — may have been more challenging for respondents without specialized knowledge. The survey did not allow enough detail to consider issues such as number of servings per week, offering foods versus serving foods (ie, what students selected), or how much food was consumed. The current study did not assess consumption or plate waste.

Our survey data indicated that the quality of elementary school lunches improved significantly between 2006–2007 and 2013–2014. Regular availability of fresh fruits, vegetables, whole grains and more healthful pizzas increased, and the availability of high-fat items such as fried potatoes, regular pizza, and high-fat milks decreased. Changes were not uniform across the nation, but many regional disparities disappeared by 2013–2014. Nevertheless, even in 2013–2014, fresh fruit was less likely to be available at majority-black or majority-Latino schools, and schools serving many socioeconomically disadvantaged students were less likely to offer salads; these demographic disparities warrant attention. Furthermore, at many schools, there is still room for improvement in providing healthful foods regularly. Supporting the implementation of salad bars in socioeconomically disadvantaged schools may be a key strategy for increasing access to healthful options for all students. Our study demonstrates the power and the value of national policy actions, such as the Healthy, Hunger-Free Kids Act, for improving school nutrition environments and supporting the health of all US schoolchildren. It is crucial that policy makers at the national, state, and local levels continue to support such actions and that funding be available for implementation and sustainability of healthful school environments.
